# Supplementary material for: Manufacturer invasion and online sales mode strategy considering the level of service quality
Source: PLoS One. 2023 Oct 17;18(10):e0292736. doi: 10.1371/journal.pone.0292736 (PMC10581468; doi:10.1371/journal.pone.0292736)
Supplement: S1 Appendix — (DOCX) [file pone.0292736.s001.docx]

**S1 Appendix**

**Proof of lemma 1**

In scenario R, due to the absence of competitive product SB, i.e., $D_{S}=0$,we have$D_{N}=\theta+\lambda s_{N}-p_{N}$. Using the backward induction method, we first differentiate the function $\pi_{E}$ with respect to $p_{N}$ to obtain the second-order derivative, which is found to be less than 0, indicating the existence of a maximum value. By differentiating the function $\pi_{E}$ with respect to $p_{N}$(i.e., $\frac{\partial\pi_{E}}{\partial p_{N}}=0$), we can obtain $p_{N}$. Similarly, based on $\pi_{B}$, we can obtain $z_{N}$. Further solving leads to the value of $w=\frac{\theta+s_{N}\lambda}{2}$. Substituting $w$ into the functions $z_{N}$, $p_{N}$, $\pi_{C}$, and $\pi_{B}$ yields the equilibrium results of Lemma 1.

**Proof of lemma 2**

The proof process is similar to Lemma 1 and further elaboration is omitted.

**Proof of lemma 3**

Using the method of reverse induction, the Hessian matrix of variable $\pi_{E}$ with respect to variables $p_{N}$ and $p_{S}$ is computed, yielding $H1=\left[\begin{matrix} -2\left(1+\gamma\right) & 2\gamma\\ 2\gamma& -2\left(1+\gamma\right) \end{matrix}\right]$. Due to $\gamma>0\text{、 }\left|H1\right|=4+8\gamma>0$, It is easy to know that the matrix *H*1 is negative definite, indicating the existence of a maximum value. Therefore, let $\frac{\partial\pi_{E}}{\partial p_{N}}=0$ and $\frac{\partial\pi_{E}}{\partial p_{S}}=0$, solving the equations yields

$p_{N}=\frac{z_{N}+\theta+s_{N}\lambda+2\left(z_{N}+\theta\right)\gamma+\left(s_{N}+s_{S}\right)\lambda\gamma}{2+4\gamma}$ and $p_{S}=\frac{z_{S}+\theta+s_{S}\lambda+2\left(z_{S}+\theta\right)\gamma+\left(s_{N}+s_{S}\right)\lambda\gamma}{2+4\gamma}$.

Furthermore, since$\frac{\partial^{2}\pi_{B}}{\partial z_{N}^{2}}=-1-\gamma<0$, it demonstrates that the function $\pi_{B}$ has a maximum point. Thus, we can use the same solution by maximizing the brand manufacturer’s profit $\pi_{B}$to obtain $z_{N}=\frac{w+\theta+s_{N}\lambda+w\gamma+z_{S}\gamma}{2+2\gamma}$.

Finally, taking the second-order partial derivatives of $\pi_{C}$ with respect to $z_{S}$ and $w$, we obtain the Hessian matrix $H2=\left[\begin{matrix} -\frac{2+\gamma\left(4+\gamma\right)}{2\left(1+\gamma\right)} & \frac{\gamma}{2} \\ \frac{\gamma}{2} & \frac{1}{2\left(-1-\gamma\right)} \end{matrix}\right]$ and $\left|H2\right|=\frac{1}{2}+\gamma>0$.

It is evident that the Hessian matrix *H*2 is negative definite, indicating the existence of a maximum value. Taking the first-order partial derivatives of $\pi_{C}$ with respect to $z_{S}$ and $w$, and setting them equal to zero, we can solve that $z_{S}=\frac{\theta+2\theta\gamma+\lambda\left(s_{S}+\left(s_{N}+s_{S}\right)\gamma\right)}{2+4\gamma}$ and $w=\frac{\theta+2\theta\gamma+\lambda\left(s_{N}+\left(s_{N}+s_{S}\right)\gamma\right)}{2+4\gamma}$.

By substituting the $z_{S}$ and $w$into $z_{N}$,$p_{N},p_{S}$,$\pi_{C}$and$\pi_{B}$we can obtain the equilibrium results of Lemma 3.

**Proof of lemma 4**

Similar to the proof of lemma 3, first, taking the second-order partial derivative of $\pi_{E}$ with respect to$p_{S}$, we obtain that $\frac{\partial^{2}\pi_{E}}{\partial p_{S}^{2}}=-2\left(1+\gamma\right)<0$. Therefore, the function$\pi_{E}$ is concave, implying the existence of a maximum value. Let $\frac{\partial\pi_{E}}{\partial p_{S}}=0$, we can obtain $p_{S}=\frac{z_{S}+\theta+s_{S}\lambda+\left(p_{N}+fp_{N}+z_{S}\right)\gamma}{2\left(1+\gamma\right)}$. Similarly, let's take the second derivative of $\pi_{B}$with respect to $p_{N}$, the result$\frac{\partial^{2}\pi_{B}}{\partial p_{N}^{2}}=\frac{\left(1-f\right)\left(\gamma\left(\left(f-1\right)\gamma-4\right)-2\right)}{1+\gamma}<0$ owing to $f<1$ and $0<\gamma<1$. Hence the function$\pi_{E}$ is also concave and exists a maximum value. Let $\frac{\partial\pi_{B}}{\partial p_{N}}=0$, we can yield $p_{N}=\frac{w\left(2+\gamma\left(4+\gamma-f\gamma\right)\right)-\left(f-1\right)\left(2s_{N}\lambda\left(1+\gamma\right)+\theta\left(2+3\gamma\right)+\gamma\left(z_{S}+s_{S}\lambda+z_{S}\gamma\right)\right)}{2\left(f-1\right)\left(\gamma\left(\left(f-1\right)\gamma-4\right)-2\right)}$.

Since the Hessian matrix of variable $\pi_{C}$ with respect to variables $w$ and $z_{S}$

$H3=\left[\begin{matrix} \frac{\left(1+\gamma\right)\left(\gamma\left(\left(f-1\right)\gamma-8\right)-4\right)}{4-2\gamma\left(\left(f-1\right)\gamma-4\right)} & \frac{\gamma}{2} \\ \frac{\gamma}{2} & \frac{2+\gamma\left(4+\gamma-f\gamma\right)}{2\left(f-1\right)\left(1+\gamma\right)} \end{matrix}\right]$.

And it’s easy to determined that *H*3 is a negative definite matrix, indicating the existence of a maximum value of $\pi_{C}$. Setting $\frac{\partial\pi_{C}}{\partial w}=0$and $\frac{\partial\pi_{C}}{\partial z_{S}}=0$, we can obtain

$z_{S}=\frac{s_{S}\lambda-\theta\left(1+2\gamma\right)\left(\left(f-1\right)\gamma-1\right)+\lambda\gamma\left(2s_{S}-\left(f-1\right)\left(s_{N}+\left(s_{N}+s_{S}\right)\gamma\right)\right)}{2\left(1+\gamma\right)\left(1+2\gamma\right)}$,$w=\frac{\left(1-f\right)\left(\theta+2\theta\gamma+\lambda\left(s_{N}+\left(s_{N}+s_{S}\right)\gamma\right)\right)}{2+4\gamma}$.

By substituting the $z_{S}$ and $w$into$p_{N},p_{S}$,$\pi_{C}$and$\pi_{B}$.

And $\pi_{C}=\frac{\left\{\begin{matrix} \theta^{2}\left(1+2\gamma\right)\left(8-4f+4\left(7-5f\right)\gamma+\left(f-1\right)\left(4f-29\right)\gamma^{2}+8\left(f-1\right)^{2}\gamma^{3}\right)+ \\ 4\left(f-1\right)s_{N}^{2}\lambda^{2}\left(1+\gamma\right)^{2}\left(\gamma\left(\left(f-1\right)\gamma-2\right)-1\right)+4\left(f-1\right)s_{N}\theta\lambda\left(1+\gamma\right)\left(1+2\gamma\right) \\ \left(\gamma\left(2\left(f-1\right)\gamma-5\right)-2\right)+2s_{S}\lambda(2\left(f-1\right)s_{N}\lambda\gamma\left(1+\gamma\right)\left(2\gamma\left(\left(f-1\right)\gamma-3\right)-3\right)+ \\ \theta\left(1+2\gamma\right)\left(4+\gamma\left(14-6f-15\left(f-1\right)\gamma+4\left(f-1\right)^{2}\gamma^{2}\right)\right))+s_{S}^{2}(16\left(1+\gamma\right)\left(1+2\gamma\right) \\ \left(\gamma\left(\left(f-1\right)\gamma-4\right)-2\right)+\lambda^{2}\left(4+\gamma\left(16+\gamma\left(25-9f-18\left(f-1\right)\gamma+4\left(f-1\right)^{2}\gamma^{2}\right)\right)\right)) \end{matrix}\right\}}{16\left(1+\gamma\right)\left(1+2\gamma\right)\left(2-\gamma\left(\left(f-1\right)\gamma-4\right)\right)}=\pi_{C}^{*}$

Thus, we can obtain the equilibrium results of Lemma 4.

**Proof of lemma 5**

First, we calculate the second-order partial derivative of $\pi_{E}$with respect to$p_{N}$ to determine the existence of a maximum value. The result is $\frac{\partial^{2}\pi_{E}}{\partial p_{N}^{2}}=-2\left(1+\gamma\right)<0$. This demonstrates that the function $\pi_{E}$ is concave, indicating the presence of a maximum value. Setting $\frac{\partial\pi_{E}}{\partial p_{N}}=0$and getting the solution of $p_{N}$,then substitute $p_{N}$into other functions like $\pi_{C}$and $\pi_{B}$. Next, we calculate the second-order partial derivative of $\pi_{B}$with respect to $z_{N}$, resulting in$\frac{\partial^{2}\pi_{B}}{\partial z_{N}^{2}}=-1-\gamma<0$ . This indicates that the function$\pi_{B}$ is also concave, affirming the existence of a maximum value. Setting $\frac{\partial\pi_{B}}{\partial z_{N}}=0$ obtain $z_{N}=\frac{w+\theta+s_{N}\lambda+\left(p_{S}-fp_{S}+w\right)\gamma}{2\left(1+\gamma\right)}$.Then, compute the Hessian matrix of variable $\pi_{C}$ with respect to variables $w$ and $p_{S}$

$$H4=\left[\begin{matrix} \frac{\left(1-f\right)\left(\gamma\left(\left(f-1\right)\gamma-8\right)-4\right)}{2\left(1+\gamma\right)} & \frac{1}{2\left(1-f\right)\gamma} \\ \frac{1}{2\left(1-f\right)\gamma} & \frac{1}{2\left(-1-\gamma\right)} \end{matrix}\right]$$

And *H*4 is a negative definite matrix, indicating the existence of a maximum value of $\pi_{C}$. Setting $\frac{\partial\pi_{C}}{\partial w}=0$and $\frac{\partial\pi_{C}}{\partial p_{S}}=0$, we can obtain

$p_{S}=\frac{\theta+2\theta\gamma+\lambda\left(s_{S}+\left(s_{N}+s_{S}\right)\gamma\right)}{2+4\gamma}$,$w=\frac{\theta\left(1+2\gamma\right)\left(1+\left(1-f\right)\gamma\right)+\lambda\left(s_{N}-\left(f-1\right)s_{S}\gamma\left(1+\gamma\right)+s_{N}\gamma\left(2+\gamma-f\gamma\right)\right)}{2\left(1+\gamma\right)\left(1+2\gamma\right)}$

By substituting the $p_{S}$ and $w$into$p_{N},z_{N}$,$\pi_{C}$and$\pi_{B}$we can obtain the equilibrium results of Lemma 5.

**Proof of lemma 6**

Using the method of reverse induction, calculate the second-order partial derivative of $\pi_{B}$with respect to$p_{N}$ determining the existence of a maximum value. The result is $\frac{\partial^{2}\pi_{B}}{\partial p_{N}^{2}}=2\left(f-1\right)\left(1+\gamma\right)<0$. This demonstrates that the function $\pi_{B}$ is concave, indicating the presence of a maximum value. Setting $\frac{\partial\pi_{B}}{\partial p_{N}}=0$and getting the solution of $p_{N}$. By taking the second partial derivatives of$\pi_{C}$ with respect to $w$and $p_{S}$, we obtain the Hessian matrix *H*5,

$H5=\left[\begin{matrix} \frac{\left(f-1\right)\left(2+\gamma\left(4+\gamma\right)\right)}{1+\gamma} & \gamma\\ \gamma& \frac{1+\gamma}{f-1} \end{matrix}\right]$.

It is evident that the Hessian matrix is negative definite, indicating the existence of a maximum value. Setting $\frac{\partial\pi_{C}}{\partial w}=0$and $\frac{\partial\pi_{C}}{\partial p_{S}}=0$ we can obtain

$p_{S}=\frac{\theta+2\theta\gamma+\lambda\left(s_{S}+\left(s_{N}+s_{S}\right)\gamma\right)}{2+4\gamma}$,$w=\frac{\left(1-f\right)\left(\theta+2\theta\gamma+\lambda\left(s_{N}+\left(s_{N}+s_{S}\right)\gamma\right)\right)}{2+4\gamma}$

By substituting the $p_{S}$ and $w$into$p_{N}$,$\pi_{C}$and$\pi_{B}$we can obtain the equilibrium results of Lemma 6.

**Proof of proposition 1**

For the contract manufacturer, when the brand manufacturer adopts the reselling strategy, we compare the demand for product SB in the RA and RR scenarios.

$\begin{matrix} D_{S}^{RA}=\frac{3s_{N}\lambda\gamma+4s_{S}\lambda\left(1+\gamma\right)+\theta\left(4+7\gamma\right)}{8\left(1+\gamma\right)} \end{matrix}$and $\begin{matrix} D_{S}^{RR}=\frac{s_{N}\lambda\gamma+2s_{S}\lambda\left(1+\gamma\right)+\theta\left(2+3\gamma\right)}{8\left(1+\gamma\right)} \end{matrix}$

Since all parameters in the model are greater than zero, it is easy to see that$D_{S}^{RR}<D_{S}^{RA}$.

When the brand manufacturer chooses the agency mode,

$\begin{matrix} D_{S}^{AA}=\frac{s_{N}\lambda\gamma+2s_{S}\left(1+\gamma\right)+\theta\left(2+3\gamma\right)}{4\left(1+\gamma\right)} \end{matrix}$,$\begin{matrix} D_{S}^{AR}=\frac{2\left(\left(f-5\right)\theta+\left(f-1\right)s_{N}\lambda-4s_{S}\lambda\right)\gamma+\left(f-1\right)\left(5\theta+2s_{N}\lambda+3s_{S}\lambda\right)\gamma^{2}-4\left(\theta+s_{S}\lambda\right)}{\left(8\left(\gamma\left(\left(f-1\right)\gamma-4\right)-2\right)\right)} \end{matrix}$

And

$$\begin{matrix} D_{S}^{AA}-D_{S}^{AR}=\frac{\left\{\begin{matrix} s_{S}\lambda\left(1+\gamma\right)\left(\gamma\left(\left(f-1\right)\gamma-8\right)-4\right)-2\left(1+f\right)s_{N}\lambda\gamma\left(1+2\gamma\right) \\ +\theta\gamma\left(-\gamma\left(13+\gamma\right)+f\left(\left(\gamma-3\right)\gamma-2\right)-14\right)-4\theta\end{matrix}\right\}}{8\left(1+\gamma\right)\left(\gamma\left(\left(f-1\right)\gamma-4\right)-2\right)} \end{matrix}$$

Given that all parameters of $D_{S}^{AA}-D_{S}^{AR}$ are greater than zero and all parameters except $\theta\text{，}$ are less than one, it is easy to obtain that in the expression of $D_{S}^{AA}-D_{S}^{AR}$ both the numerator and denominator are always less than zero. Therefore, $D_{S}^{AA}-D_{S}^{AR}$ is always greater than zero.

Similarly, in the case of the brand manufacturer, the comparison between $D_{N}^{RA}=\frac{1}{8}\left(\theta+s_{N}\lambda\right)$ and $D_{N}^{AA}=\frac{1}{4\left(\theta+s_{N}\lambda\right)}$ reveals that $D_{N}^{RA}>D_{N}^{AA}$.

$D_{N}^{RR}=\frac{\theta+s_{N}\lambda}{8}$,$D_{N}^{AR}=\frac{s_{S}\lambda\gamma+2s_{N}\lambda\left(1+\gamma\right)+\theta\left(2+3\gamma\right)}{8\left(1+\gamma\right)}$and $D_{N}^{AR}-D_{N}^{RR}=\frac{\theta+2\theta\gamma+\lambda\left(s_{N}+\left(s_{N}+s_{S}\right)\gamma\right)}{8\left(1+\gamma\right)}>0$

Hence, it can be concluded that $D_{N}^{AR}>D_{N}^{RR}$, thereby the **Proposition 1** is proven.

**Proof of proposition 2**

In the brand manufacturer's reselling mode, compare the magnitudes of $\pi_{C}$ in the benchmark scenario R, scenario RA, and scenario RR.

$\begin{matrix} \text{π}_{\text{C}}^{\text{RR}}-\text{π}_{\text{C}}^{\text{R}}=\frac{\left\{\begin{matrix} 2s_{N}\theta\lambda\gamma\left(1+2\gamma\right)+4s_{S}\lambda\left(1+\gamma\right)\left(\theta+2\theta\gamma+s_{N}\lambda\gamma\right)-s_{N}^{2}\lambda^{2}\gamma\\ +2s_{S}^{2}\left(1+\gamma\right)\left(\lambda^{2}-8+\left(\lambda^{2}-16\right)\gamma\right)+\theta^{2}\left(2+\gamma\left(7+6\gamma\right)\right) \end{matrix}\right\}}{16\left(1+\gamma\right)\left(1+2\gamma\right)} \end{matrix}$,

$\text{π}_{\text{C}}^{\text{RA}}-\text{π}_{\text{C}}^{\text{R}}=\left\{\begin{matrix} \frac{1}{16}(2\theta\lambda\left(\frac{\left(3-4f\right)s_{N}\gamma}{1+\gamma}-4\left(f-1\right)s_{S}\right)+\frac{\theta^{2}\left(4+7\gamma-4f\left(1+2\gamma\right)\right)}{1+\gamma} \\ +\frac{1}{\left(1+\gamma\right)\left(1+2\gamma\right)}(s_{N}^{2}\lambda^{2}\gamma\left(\left(2-4f\right)\gamma-1\right)-8\left(f-1\right)s_{N}s_{S}\lambda^{2}\gamma\left(1+\gamma\right) \\ -4s_{S}^{2}\left(1+\gamma\right)\left(4+8\gamma+\left(f-1\right)\lambda^{2}\left(1+\gamma\right)\right))) \end{matrix}\right\}$,

Assuming that$\theta_{1}\text{，}\theta_{2}$are positive solutions of $\text{π}_{\text{C}}^{\text{RR}}-\text{π}_{\text{C}}^{\text{R}}=0$ and $\text{π}_{\text{C}}^{\text{RA}}-\text{π}_{\text{C}}^{\text{R}}=0$ with respect to $\theta$ respectively, it can be easily observed that both $\text{π}_{\text{C}}^{\text{RA}}-\text{π}_{\text{C}}^{\text{R}}$ and $\text{π}_{\text{C}}^{\text{RA}}-\text{π}_{\text{C}}^{\text{R}}$ are quadratic expressions with respect to $\theta$. Furthermore, the quadratic coefficient of $\theta$ is greater than 0, as well as the linear coefficient of $\theta$. Hence, when $\theta>\theta_{1}\text{，π}_{\text{C}}^{\text{RR}}-\text{π}_{\text{C}}^{\text{R}}>0$; and when $\theta>\theta_{2}\text{，π}_{\text{C}}^{\text{RA}}-\text{π}_{\text{C}}^{\text{R}}>0$.

Consequently, when $\theta>\hat{\theta}^{R}$,$\hat{\theta}^{R}=\max\left\{\theta_{1},\theta_{2}\right\}$,it turns out that $\text{π}_{\text{C}}^{\text{RA}}-\text{π}_{\text{C}}^{\text{R}}>0$ and $\text{π}_{\text{C}}^{\text{RR}}-\text{π}_{\text{C}}^{\text{R}}>0$.Thus the contract manufacturer will always choose to engage in intrusion in this situation.

when the brand manufacturer adopts agency mode, we compare $\text{π}_{\text{C}}^{\text{A}}$ in scenario A, $\text{π}_{\text{C}}^{\text{AR}}$ in scenario AR, and $\text{π}_{\text{C}}^{\text{AA}}$ in scenario AA. It is evident that:

$\text{π}_{\text{C}}^{\text{AR}}-\text{π}_{\text{C}}^{\text{A}}=\frac{\left\{\begin{matrix} \frac{1}{8}\left(f-1\right)\left(\theta+s_{N}\lambda\right)^{2}-(\theta^{2}\left(1+2\gamma\right)(8-4f+4\left(7-5f\right)\gamma+\left(f-1\right)\left(4f-29\right)\gamma^{2}+ \\ 8\left(f-1\right)^{2}\gamma^{3})+4\left(f-1\right)s_{N}^{2}\lambda^{2}\left(1+\gamma\right)^{2}\left(\gamma\left(\left(f-1\right)\gamma-2\right)-1\right)+4\left(f-1\right)s_{N}\theta\lambda\left(1+\gamma\right) \\ \left(1+2\gamma\right)\left(\gamma\left(2\left(f-1\right)\gamma-5\right)-2\right)+2s_{S}\lambda(2\left(f-1\right)s_{N}\lambda\gamma\left(1+\gamma\right)\left(2\gamma\left(\left(f-1\right)\gamma-3\right)-3\right) \\ +\theta\left(1+2\gamma\right)\left(4+\gamma\left(14-6f-15\left(f-1\right)\gamma+4\left(f-1\right)^{2}\gamma^{2}\right)\right))+s_{S}^{2}(16\left(1+\gamma\right)\left(1+2\gamma\right) \\ \left(\gamma\left(\left(f-1\right)\gamma-4\right)-2\right)+\lambda^{2}\left(4+\gamma\left(16+\gamma\left(25-9f-18\left(f-1\right)\gamma+4\left(f-1\right)^{2}\gamma^{2}\right)\right)\right))) \end{matrix}\right\}}{16\left(1+\gamma\right)\left(1+2\gamma\right)\left(\gamma\left(\left(f-1\right)\gamma-4\right)-2\right)}$,

$\text{π}_{\text{C}}^{\text{AA}}-\text{π}_{\text{C}}^{\text{A}}=\frac{\left\{\begin{matrix} \left(1-f\right)\theta^{2}\left(2+3\gamma\right)-2\left(f-1\right)\theta\lambda\left(s_{N}\gamma+2s_{S}\left(1+\gamma\right)\right)+\frac{\left(f-1\right)s_{N}^{2}\lambda^{2}\gamma}{1+2\gamma} \\ +\frac{4\left(1-f\right)s_{N}s_{S}\lambda^{2}\gamma\left(1+\gamma\right)-2s_{S}^{2}\left(1+\gamma\right)\left(4+8\gamma+\left(f-1\right)\lambda^{2}\left(1+\gamma\right)\right)}{1+2\gamma} \end{matrix}\right\}}{8\left(1+\gamma\right)}$,

Similarly, assuming that$\theta_{3}$and$\theta_{4}$ are positive solutions of $\text{π}_{\text{C}}^{\text{AR}}-\text{π}_{\text{C}}^{\text{A}}=0$ and $\text{π}_{\text{C}}^{\text{AA}}-\text{π}_{\text{C}}^{\text{A}}=0$ with respect to $\theta$ respectively, it can be easily observed that both $\text{π}_{\text{C}}^{\text{AR}}-\text{π}_{\text{C}}^{\text{A}}$ and $\text{π}_{\text{C}}^{\text{AA}}-\text{π}_{\text{C}}^{\text{A}}$ are quadratic expressions with respect to $\theta$. Furthermore, the quadratic coefficient of $\theta$ is greater than 0, as well as the linear coefficient of $\theta$. Hence, when $\theta>\theta_{3}\text{，π}_{\text{C}}^{\text{AR}}-\text{π}_{\text{C}}^{\text{A}}>0$; and when $\theta>\theta_{4}$,it shows $\text{π}_{\text{C}}^{\text{AA}}-\text{π}_{\text{C}}^{\text{A}}>0$ .

Consequently, when $\theta>\hat{\theta}^{A}$,$\hat{\theta}^{A}=\max\left\{\theta_{3},\theta_{4}\right\}$,it turns out that $\text{π}_{\text{C}}^{\text{AR}}-\text{π}_{\text{C}}^{\text{A}}>0$ and $\text{π}_{\text{C}}^{\text{AA}}-\text{π}_{\text{C}}^{\text{A}}>0$. And the contract manufacturer will always choose to engage in intrusion in this situation.

**Proof of proposition 3**

When the brand manufacturer adopts the reselling mode,

$\begin{matrix} \text{π}_{\text{C}}^{\text{RR}}-\text{π}_{\text{C}}^{\text{RA}}=\frac{\left(2f-1\right)\left(\theta+2\theta\gamma+\lambda\left(s_{S}+\left(s_{N}+s_{S}\right)\gamma\right)\right)^{2}}{8\left(1+\gamma\right)\left(1+2\gamma\right)} \end{matrix}$.

Because $\gamma>0$, from Equation $\text{π}_{\text{C}}^{\text{RR}}-\text{π}_{\text{C}}^{\text{RA}}$, it can be derived that the invasion threshold $\bar{f}^{R}$ of the contract manufacturer with respect to parameter $f$ is equal to 0.5. So when$f<\bar{f}^{R}$and $\text{π}_{\text{C}}^{\text{RR}}-\text{π}_{\text{C}}^{\text{RA}}<0$, the contract manufacturer chooses the agency mode. When$f>\bar{f}^{R}$and$\text{π}_{\text{C}}^{\text{RR}}-\text{π}_{\text{C}}^{\text{RA}}>0$ , the contract manufacturer chooses the reselling mode.

When the brand manufacturer adopts the agency mode,

$\begin{matrix} \text{π}_{\text{C}}^{\text{AR}}-\text{π}_{\text{C}}^{\text{AA}}=\frac{\left\{\begin{matrix} (4\left(1-2f\right)\left(\theta+s_{S}\lambda\right)^{2}-4\left(\theta+s_{S}\lambda\right)\left(\left(5f-3\right)\theta+\left(\left(f-1\right)s_{N}-2s_{S}+4fs_{S}\right)\lambda\right)\gamma\\ +\left(f-1\right)\left(\begin{matrix} \left(2f-9\right)\theta^{2}-2\left(2\left(2+f\right)s_{N}+\left(5-4f\right)s_{S}\right)\theta\lambda\\ -\left(2\left(f-1\right)s_{N}^{2}+4s_{N}s_{S}+\left(3-4f\right)s_{S}^{2}\right)\lambda^{2} \end{matrix}\right)\gamma^{2} \end{matrix}\right\}}{16\left(1+\gamma\right)\left(\gamma\left(\left(f-1\right)\gamma-4\right)-2\right)} \end{matrix}$.

It can be observed that the numerator of expression $\text{π}_{\text{C}}^{\text{AR}}-\text{π}_{\text{C}}^{\text{AA}}$ is a quadratic expression with respect to$f$, and the coefficient of the quadratic term of $f$ is positive.(i.e., the coefficient in front of $f^{2}$ is positive). Another solution for$\text{π}_{\text{C}}^{\text{AR}}-\text{π}_{\text{C}}^{\text{AA}}=0$is $f_{1}<0$, and $\left.\frac{\partial^{2}\text{π}_{\text{C}}^{\text{AR}}-\text{π}_{\text{C}}^{\text{AA}}}{\partial f^{2}}\right|_{f=\bar{f}^{A}}>0$. Thus, when $f<\bar{f}^{A},and\text{ π}_{\text{C}}^{\text{AR}}-\text{π}_{\text{C}}^{\text{AA}}<0$, the contract manufacturer chooses the agency mode. When $f>\bar{f}^{A},and\text{ π}_{\text{C}}^{\text{AR}}-\text{π}_{\text{C}}^{\text{AA}}>0$, the contract manufacturer chooses the reselling mode.

Because the threshold for commission rate in the reselling mode is$\bar{f}^{R}=0.5$, we compare $\bar{f}^{A}-\bar{f}^{R}$ with zero. Although the expression for $\bar{f}^{A}$ is complex, it can be easily proven through calculations and simulations using Mathematica software that $\bar{f}^{A}-\bar{f}^{R}>0$under the conditions of parameters that $s_{N},s_{S},\gamma,\lambda\in\left(0,1\right)$ and $\theta>1$. As shown in Fig 4, the ordinate $\bar{f}$represents the threshold for commission rate, i.e., $\bar{f}^{A}$or $\bar{f}^{R}$. It can be observed that the threshold $\bar{f}^{A}$ for commission rate in the brand manufacturer's agency mode is always greater than the threshold$\bar{f}^{R}$ in the reselling mode. Therefore, Proposition 3 is established.

**Proof of proposition 4**

It is straightforward to prove that $\frac{\partial\bar{f}^{A}}{\partial s_{S}}<0$ and $\frac{\partial\bar{f}^{A}}{\partial s_{N}}>0$. And the detailed proof process is omitted.
