# Supplementary material for: Manufacturer invasion and online sales mode strategy considering the level of service quality
Source: PLoS One. 2023 Oct 17;18(10):e0292736. doi: 10.1371/journal.pone.0292736 (PMC10581468; doi:10.1371/journal.pone.0292736)
Supplement: Attachment — Submitted filename: Recommendation-PONE-D-23-21727.docx [file pone.0292736.s002.docx]

**Research on contract manufacturer invasion and sales model**

**considering the level of service quality**

This study explores the decision-making process and choice of invasion sales modes by contract manufacturers considering service quality disparities with brand manufacturers. Specifically, it constructs a three-tier supply chain system comprised of a brand manufacturer, a contract manufacturer, and an e-commerce platform. The pivotal question of whether a contract manufacturer, aspiring to build its own brand, should establish a brand and select an appropriate sales model on e-commerce platforms becomes the focus of this research. Despite the author's substantial efforts, I believe from a professional standpoint that this paper does not yet meet the publication standards of the PLOS ONE journal at this stage. Therefore, I recommend a "**Major Revision**" decision and provide the following specific review comments:

1. The introduction lacks an explanation of service quality and fails to articulate the paper's innovative aspects and the arrangement of its structure. The literature review section that states "this study aims to investigate the following issues" should also be included in the introduction.

2. The literature review only provides a summary of related streams, and the referenced literature is simply listed without logical connections between them. The citation format is inconsistent, and there are several inaccuracies in the page numbers of the references in the References section that need to be corrected. The review of the literature is overly broad and does not showcase the theoretical value of existing research or how this study extends and builds upon related literature, nor the theoretical contribution of this paper to the research gap.

3. The selection of parameters in the example analysis lacks necessary real-world support. The example graphs lack necessary details (such as annotation explaining thresholds), and the majority of them are analyzing the commission rate *f*.

4. The conclusions are overly convoluted. Among the five conclusions in this paper, only the fourth one mentions service quality. The management applications are also overly broad and do not closely relate to the previous analysis and conclusions.
