# Supplementary material for: Manufacturer invasion and online sales mode strategy considering the level of service quality
Source: PLoS One. 2023 Oct 17;18(10):e0292736. doi: 10.1371/journal.pone.0292736 (PMC10581468; doi:10.1371/journal.pone.0292736)
Supplement: Attachment — Submitted filename: comments for plosone01.docx [file pone.0292736.s003.docx]

The study examines the contract manufacturer's invasion against downstream market and its selling mode selection with the consideration of the differences in service quality levels. However, there are still many problems in the writing and modeling of this submission. Thus, I recommend to have a major revision or resubmission.

The problems are as follows:

First, “online selling mode strategies” seems to be suitable for “sales model” in title. In addition, words like "research on" or "study of" do not often appear in the title. please check it.

Second, service quality is a very important point in this article. Why is it not explained in the introduction and what are its practical application scenarios?

Third, the size of *s_n_* and *s_S_* needs to be explained based on real cases. The examples of Apple and Samsung does not seem to illustrate this point. Is Apple's mobile phone better than Samsung's Galaxy series? I think it cannot be simply defined. Samsung is just a supplier of iPhone screens, and the quality of terminal products cannot be defined by a single LED screen. Could you please list some more examples. What does service quality specifically refer to? Is service quality the difference between brands? According to the author's example, I believe that service quality should be referred to as "brand effect".

Fourth, *Π*_C_=*wD_N_* is always a non-negative number, and the profit function of brand manufacturer always be smaller than the upstream, which is definitely unreasonable and unrealistic. Regardless of whether the contract manufacturer invades the downstream market or not, I think that its production should have a cost. In addition, after the contract supplier invades the downstream market, its profits are only high but not low, and the cost of service quality is low given by *s_s_*<s_N_. we can always obtain *Π*_C_>*Π*_N_. Compared to not invading, contract manufacturer’s invasion profits are definitely greater than its brand merchants. In order to occupy the market, contract manufacturers will definitely invade downstream markets. This is a result that can be drawn without proof. Therefore, I have doubts about the profit function of upstream and downstream manufacturers.

Fifth, as for conducting numerical analysis, why do *s_s_* and *s_N_* are less than 1? The range of *s* defined in 3.1 Demand function is greater than 0. its square is taken as the quality cost, which is generally less than 1. Would this cost be a bit small?

Sixth, check the grammar of the paper carefully. The language logic and grammar should be considered carefully and comprehensively. A professional proof-reading is mandatory. For example, in Abstract “The equilibrium profits under different sales mode combinations are determined using reverse ...” Is there a preposition missing before 'using'?
