# Supplementary material for: Manufacturer invasion and online sales mode strategy considering the level of service quality
Source: PLoS One. 2023 Oct 17;18(10):e0292736. doi: 10.1371/journal.pone.0292736 (PMC10581468; doi:10.1371/journal.pone.0292736)
Supplement: Attachment — Submitted filename: Response to Reviewers.docx [file pone.0292736.s004.docx]

Response Letter to PLOS ONE Submission

Paper ID: PONE-D-23-21727

Paper Title: Manufacturer invasion and online sales mode strategy considering the level of service quality

Dear Editors and Reviewers:

It is with excitement that I resubmit to you a revised version of manuscript“Manufacturer invasion and online sales mode strategy considering the level of service quality”for the“PLOS ONE”. We thank the review team for the positive feedback on the revision and the opportunity to publish our research. The review team has been extremely helpful in providing constructive suggestions. We are truly grateful for all the excellent advice we have received. Thank you very much!

We have substantially revised our manuscript providing details in the current version. We submitted 3 files, including a revised manuscript “Revised Manuscript with Track Changes”, An unmarked version of your revised paper without tracked changes file labeled “Manuscript” and the response letter file labeled “Response to Reviewers”. Furthermore, we have also updated the figures and author contributions within the manuscript**.** We hope that the revision is acceptable and look forward to hearing from you soon.

(RC: Reviewer’s Comment, AR: Author's Response)

With best wishes.

Sincerely Yours,

Guiping Su

Address correspondence to: ebsugpcassia@mail.scut.edu.cn

In the following, we respond to each of your concerns and recommendations. We have formatted the reviewer's comments in bold italics for easier distinction in our responses. "RC" signifies the reviewer's comment and "AR" signifies the author's response.

**Answers to reviewers 1**

Reviewer #1

RC1.0: ***This paper study the decision-making process of self-brand intrusion by CMs and the choice of sale modes. The research problem is interesting, the model is solid, and the results seems right. I appreciate the authors’ hard work. I propose the following issues, which need the authors’ concerns***.

AC1.0: We thank the reviewer for the kind comments.

RC1.1: ***The Introduction is too short, such that the motivation, the*** ***scientific problem and the contribution of this paper is not clearly.***

AR1.1: Thank you for your suggestion, according to your suggestion, we added the information you suggested. We have augmented the introduction with a description of the background and motivation for this study, and in the introduction and literature review sections, we have respectively expounded upon the theoretical contributions and practical contributions of this study. The specific scientific problem can be observed in the penultimate paragraph of the Introduction The added information is as follows (see details in lines 11-22 of the revised manuscript introduction section).

RC1.2: ***In Lemma 1, why the resulting $\Pi_{C}$ and $\Pi_{B}$ are functions of $f$? I guess this is a typo.***

AR1.2: Thank you for bringing this typographical error to our attention. We sincerely appreciate your meticulous review, which has been immensely helpful in improving the quality of our manuscript. We have promptly made the necessary corrections as per your suggestions and have thoroughly reviewed the entire article to ensure the absence of similar errors. Your feedback has been invaluable, and we are grateful for your time and effort in reviewing our work.

RC1.3: ***In Lemma 4, the*** ***mathematical symbols are ugly. The authors should modify them.***

AR1.3: We sincerely apologize for the complexity introduced by the mathematical symbols in Lemma 4. We have made revisions to simplify the lengthy formulas, resulting in a more concise presentation. Specifically, we have replaced the overly complicated mathematical symbol $\pi_{C}$ in lemma 4 with $\pi_{C}^{*}$ and provided its detailed expression in the appendix for clarity. Given that our study addresses a three-tier supply chain structure, which includes an additional layer compared to traditional two-tier structures, and involves numerous parameters, many of the formulas involved in this study tend to be intricate. We have endeavored to simplify these formulas to enhance the overall neatness of the manuscript. If you believe that further simplification of mathematical symbols is advisable, we would greatly appreciate your guidance. We are fully committed to making any necessary adjustments to improve the readability of our manuscript.

RC1.4: ***I would like to ask why the authors submit this paper to Plos One? It seems that the paper is more suitable to a business journal.***

AR1.4: Thank you for your inquiry. Firstly, PLOS ONE is a reputable journal with a team of distinguished editors and reviewers, including yourself, committed to upholding rigorous standards in manuscript evaluation. PLOS ONE holds the distinction of being recognized as a Web of Science Core Journal, with an impressive 5-year impact factor of 3.8. We humbly aspire to have our research published in this prestigious journal.

Secondly, PLOS ONE is a comprehensive journal that encompasses articles from different research fields. After conducting a thorough literature review, we've identified relevant publications in related fields within PLOS ONE, such as Li et al. [1]and Yang et al. [2], etc. And these articles were published in recent years. Given the presence of relevant research in similar domains, we've made the decision to submit our manuscript to PLOS ONE, with the hope of earning the valuable consideration of experts like yourself.

**Answers to reviewers 2**

Reviewer #2

RC2.0: ***This study explores the decision-making process and choice of invasion sales modes by contract manufacturers considering service quality disparities with brand manufacturers. Specifically, it constructs a three-tier supply chain system comprised of a brand manufacturer, a contract manufacturer, and an e-commerce platform. The pivotal question of whether a contract manufacturer, aspiring to build its own brand, should establish a brand and select an appropriate sales mode on e-commerce platforms becomes the focus of this research. Despite the author's substantial efforts, I believe from a professional standpoint that this paper does not yet meet the publication standards of the PLOS ONE journal at this stage. Therefore, I recommend a "Major Revision" decision and provide the following specific review comments:***

AR2.0: Thank you for your careful evaluation of this manuscript. We sincerely appreciate your thoughtful and constructive feedback on our manuscript. Your comments have provided valuable insights that will help us improve the quality and relevance of our research. We greatly respect your expertise and professionalism in assessing our work. Your feedback will undoubtedly contribute to the enhancement of our manuscript.

Your observation regarding the alignment of our manuscript with the publication standards of PLOS ONE is duly noted. We understand the significance of meeting the high standards set by the journal, and we are committed to making the necessary revisions to address your concerns. Specific revisions can be found in following the responses to the questions you’ve listed.

RC2.1: ***The introduction lacks an explanation of service quality and fails to articulate the paper's innovative aspects and the arrangement of its structure. The literature review section that states "this study aims to investigate the following issues" should also be included in the introduction.***

AR2.1: Thanks for your help. We feel really sorry for our carelessness. According to your nice suggestions, we have made extensive corrections to our previous draft. 1) First, regarding the issue of the lacks an explanation of service quality in the introduction, following your advice, we have incorporated a detailed explanation of service quality along with relevant background information into the introduction. Please refer to lines 11 to 22 in the introduction section for specific details. 2) Concerning the problem of the introduction not accurately conveying the innovation of the study, we have added further relevant explanations about the research's novelty. The innovation of this research lies in its exploration of contract manufacturers' invasion through online channels, considering the crucial factor of service quality in online product sales, a topic that has been rarely addressed by scholars in the past. Please review lines 44 to 55 (begin with “Under this background…”**)** in the introduction section for specific elaboration. 3) We have also followed your advice by adding a section outlining this studies’ structure arrangement in the last paragraph of the introduction section. Additionally, as per your request, we have moved the part ***"this study aims to investigate the following issues"*** from the literature review to the article's introduction. Specifically, this can be included in the final part of the introduction. Once again, we greatly appreciate your valuable feedback.

RC2.2: ***The literature review only provides a summary of related streams, and the referenced literature is simply listed without logical connections between them. The citation format is inconsistent, and there are several*** ***inaccuracies in the page numbers of the references in the References section that need to be corrected. The review of the literature is overly broad and does not showcase the theoretical value of existing research or how this study extends and builds upon related literature, nor the theoretical contribution of this paper to the research gap.***

AR2.2: It is really against mistake to the whole quality of our study. We feel sorry for our carelessness. We sincerely appreciate the valuable feedback regarding the literature review section of our manuscript. We have taken these comments seriously and have made significant revisions including the addition of some relevant literature to address your concerns.

Firstly, we have revised the literature review to ensure a more coherent and logically connected presentation of the cited works. We sincerely apologize for the issue of inaccuracies in the page numbers of the references, which was due to oversight in the formatting settings of the reference management software. We have also carefully reviewed and corrected the citation format and page numbers in the references section to eliminate any inconsistencies and inaccuracies.

Furthermore, we have refined the literature review to focus on the specific theoretical contributions of our study and how it extends and builds upon the existing body of research in the field. We have narrowed down the scope of literature covered in the literature review to ensure that the cited literature is closely relevant to the subject of our study. In response to your suggestion regarding the theoretical value of existing research, we have added relevant discussions at the end of each paragraph of literature review section. And two closely related references to this study are the article of Chen et al. [3] and the article of Shi [4], upon which we have raise new questions concerning the choice of invasion sales modes by contract manufacturers and not just the invasion problem, and our study mostly builds upon these two related articles. Please refer to lines 80 to 96 in the literature review section for specific details(begin with “Closely related to this study are the articles…”). Additionally, to address the issue of the theoretical contribution of our paper to the research gap in the field, as per your recommendation, we have supplemented and emphasized this point in the concluding part of the literature review. We have emphasized the significance of our research in bridging the identified research gap and contributing to the theoretical development of the subject matter. We have made substantial revisions to the literature review. Please see more details in the literature review section of the revised manuscript.

RC2.3: ***The selection of parameters in the example analysis lacks necessary real-world support. The example graphs lack necessary details (such as annotation explaining thresholds), and the majority of them are analyzing the commission rate f.***

AR2.3: We adopt your suggestion. As this study primarily involves theoretical modeling, the selection of parameter values in the example analysis may not perfectly align with real-world scenarios. However, it's worth noting that the example analysis in this research draws from existing literature and thus maintains theoretical credibility. In response to the lack of necessary details in the example figures and charts, we have heeded your suggestion and added essential annotations to each chart, including the annotation explaining thresholds.

Given that the key parameters of interest in this study, specifically the commission rate ($f$) and the relative service quality level ($s_{N}$ or $s_{S}$), significantly influence decision-making, and the service quality parameters ($s_{N}$ or $s_{S}$) in turn affect the commission rate threshold, which ultimately impacts the decisions. The primary basis for decision-making in this study is the threshold commission rate. Consequently, we have devoted considerable attention to discussing the commission rate ($f$) and its threshold in this study.

RC2.4: ***The conclusions are overly convoluted. Among the five conclusions in this paper, only the fourth one mentions service quality. The management applications are also overly broad and do not closely relate to the previous analysis and conclusions.***

AR2.4: We appreciate the reviewer's feedback on our paper. We understand the concern about the clarity of our conclusions and their alignment with the rest of the paper.

Regarding the complexity of the conclusions, we acknowledge that the conclusions in our paper could benefit from further simplification and streamlining. We have revised the conclusions making our conclusions more straightforward and ensuring that they align closely with the main findings of our study.

Additionally, about the question that only the fourth conclusion mentions service quality.

with regard to the conclusions in Proposition 3 concerning the threshold value of commission rate ($f$), we have elaborated on the relationship between the threshold value of commission rate ($f$) and the service quality level in Proposition 4. Due to the complexity of the supply chain structure in this paper, the derived equilibrium result formulae are also relatively intricate, making it impractical to deduce purely mathematical conclusions regarding the impact of service quality levels. Furthermore, to emphasize the significance of service quality levels, we extensively examine the influence of service quality levels on the manufacturer's sales mode decisions by using numerical simulation in section 5 strategy discussion.

Concerning the management applications, we revise our conclusions to ensure that service quality is appropriately addressed across all relevant conclusions to maintain consistency and coherence in this study. And we refine the management applications section to ensure a closer connection with our research findings. See more details in lines 21 to 32 in the conclusions section (begin with “Based on the findings of this study…”).

We genuinely thank you for the constructive comments, which have undoubtedly improved the quality of our manuscript. We are committed to continually enhancing our work to meet the high standards of this journal, and we welcome any further guidance and feedback from you to ensure the excellence of our research.

**Answers to reviewers 3**

Reviewer #3

RC3.0: ***The study examines the contract manufacturer's invasion against downstream market and its selling mode selection with the consideration of the differences in service quality levels. However, there are still many problems in the writing and modeling of this submission. Thus, I recommend to have a major revision or resubmission.***

AR3.0: Thanks for your suggestions. We feel sorry for our poor writings. We have carefully revised the manuscript in accordance with the problems you have listed, and we sincerely hope the revised manuscript meets your approval.

RC3.1: ***First,*** ***“online selling mode strategies” seems to be suitable for “sales mode” in title. In addition, words like "research on" or "study of" do not often appear in the title. please check it.***

AR3.1: We feel sorry for the improper wording. Your suggestion really means a lot to us. Yes, it would be more suitable if we substitute “sales mode” with“online selling mode strategies” in title, and delete the words "research on" or "study of" as your concerning. The revised title is “*Manufacturer invasion and online sales mode strategy considering the level of service quality*”.

RC3.2: ***Second, service quality is a very important point in this article. Why is it not explained in the introduction and what are its practical application scenarios?***

AR3.2: Thank you for your valuable suggestions. We regret the shortcomings in our work, and based on your feedback, we have implemented revisions. We have added a detailed explanation of service quality and introduced relevant practical application scenarios in the introduction section. Please refer to the latter part of the first paragraph in the introduction for specific details. Your input has been greatly appreciated in improving our manuscript.

RC3.3: ***Third, the size of sn and sS needs to be explained based on real cases. The examples of Apple and Samsung does not seem to illustrate this point. Is Apple's mobile phone better than Samsung's Galaxy series? I think it cannot be simply defined. Samsung is just a supplier of iPhone screens, and the quality of terminal products cannot be defined by a single LED screen. Could you please list some more examples. What does service quality specifically refer to? Is service quality the difference between brands? According to the author's example, I believe that service quality should be referred to as "brand effect".***

AR3.3: Thanks for your insightful comment. We apologize for not providing a sufficiently detailed explanation of the definition of service quality levels and related examples, which led to your misunderstanding. The service quality referred to in this study arises from this background that in the current digital landscape, an increasing number of manufacturers are transitioning from merely providing products to offering a "product + service" approach. Specifically, concerning online consuming channels, product quality cannot be directly sensed; it can only be assessed and evaluated through service quality. Hence, the significance of service quality in online shopping is evident, and this is the reason of our consideration of this factor in this study.

In this context, services encompass not only post-purchase services such as repair services (which can vary between different online retailers) but also various pre-purchase activities aimed at facilitating consumer purchases. For instance, this includes the level of detail provided by hosts during online live streaming product introductions, the persuasive impact of live stream hosts on consumers, and recommendations before purchase, etc. These aspects collectively contribute to the assessment of service quality in online shopping. It's important to note that service quality can be slightly considered a differentiation factor between brands, but it should not be solely attributed to "brand effects." Instead, this differentiation arises from variances in product service quality among online manufacturers, rather than brand distinctions alone. For a more detailed explanation and additional examples, please refer to the revised Introduction section, specifically from the lines 11 to 22 and lines 26 to 36.

RC3.4: ***Fourth,*** *Π*_C_=*wD_N_* ***is always a non-negative number, and the profit function of brand manufacturer always be smaller than the upstream, which is definitely unreasonable and unrealistic. Regardless of whether the contract manufacturer invades the downstream market or not, I think that its production should have a cost. In addition, after the contract supplier invades the downstream market, its profits are only high but not low, and the cost of service quality is low given by*** *s_s_*<s_N_***. we can always obtain*** *Π*_C_>*Π*_N_***. Compared to not invading, contract manufacturer’s invasion profits are definitely greater than its brand merchants. In order to occupy the market, contract manufacturers will definitely invade downstream markets. This is a result that can be drawn without proof. Therefore, I have doubts about the profit function of upstream and downstream manufacturers.***

AR3.4: We appreciated your attention to detail. We feel great thanks for your professional review work on our article. As you are concerned, the profit of contract manufacturer, denoted as $\pi_{C}$, is always non-negative in this study. This is because, considering the complexity of supply chain structure and modeling, and for analytical tractability, the model assumes a production cost of zero. The product-related costs are normalized to be zero so that we can focus on the effects of the commission rate and service quality level, based on the existing literature Chen, Liang and Yao [3]. This assumption does not significantly impact the conclusions of the article. Even if this production cost were to be considered as a unit fixed cost, it would be transferred through the wholesale price to the brand manufacturer and then to the consumers. This effectively increases the final product's price equally, without altering the products competition intensity within the market. Additionally, the article primarily focuses on comparing the profits of brand manufacturers and contract manufacturers respectively under different sales modes. It does not aim to emphasize the difference in profits between them. The profit levels can be adjusted by including a fixed cost, which is not the primary focus of this study. The main objective of this study is to investigate the profit situations of brand manufacturers and contract manufacturers under different sales mode scenarios within the same supply chain member. The decision of whether contract manufacturers invade or not is considered in comparison to their own profit levels before invasion. The choice of sales mode is also based on a comparison of equilibrium profits under different sales mode scenarios for the same supply chain member, rather than comparing profits with other members of the supply chain. Consequently, the profit function of upstream and downstream manufacturers is derived. Once again thanks for your insightful comment.

RC3.5: ***Fifth, as for conducting numerical analysis, why do*** *s_s_* ***and*** *s_N_* ***are less than 1? The range of s defined in 3.1 Demand function is greater than 0. its*** ***square is taken as the quality cost, which is generally less than 1. Would this cost be a bit small?***

AR3.5: We feel sorry that we did not provide enough information about the service quality level parameter. We have added an additional explanation of this parameter in the model setup. The condition for the service quality level is effectively required to be greater than 0; for the convenience of section 5 numerical simulations in this study, we set the service quality level less than 1. Actually, the conclusion also holds for service quality levels greater than 1. The parameter service quality level, in conjunction with parameter $\lambda$, jointly influences the demand.

Regarding the issue of service quality cost, while this cost is relatively small, it does not affect the conclusions. We had previously considered adding a corresponding coefficient $k$ in front of its quadratic term, denoting the service quality cost as $ks_{i}^{2}(i=S,N)$.However, since this cost is a fixed cost, adjustments to the coefficient in front of the squared term would not overly impact the conclusions of this study. Additionally, adjusting this cost would increase the number of parameters in this study, making the model results more complex. Therefore, we have refrained from making extensive adjustments to this cost, and the coefficient in front of the service quality cost is 1. As a result, this cost seems relatively small.

RC3.6: ***Sixth, check the grammar of the paper carefully. The language logic and grammar should be considered carefully and comprehensively. A professional proof-reading is mandatory. For example, in Abstract “The equilibrium profits under different sales mode combinations are determined using reverse ...” Is there a preposition missing before 'using'?***

AR3.6: Thank you for your thorough review and valuable feedback. We have carefully considered and addressed the grammatical issues you pointed out. We have also undergone a comprehensive proof-reading process to enhance the language quality and overall coherence of the paper. Regarding the specific example you mentioned in the Abstract, we appreciate your keen eye for detail. The sentence has been revised to improve clarity and grammatical correctness. We have added the preposition "by" before "using" to ensure proper phrasing: "The equilibrium profits under different sales mode combinations are determined by using reverse ..."

Once again, we would like to express our gratitude for your constructive feedback, which has significantly contributed to improving the quality of our manuscript. If you have any further suggestions or concerns, please do not hesitate to let us know.

Thank you once again for your positive feedback. As elaborated in our responses, we have reviewed the paper several times and made some careful adjustments in our writing. We hope that we have communicated the essence of our work succinctly. Thank you so much for the opportunity to improve and publish our research. Please do not hesitate to contact us with any questions.

**Journal requirements:**

In response to the additional five requirements of the **Journal requirements** in the View Letter, we have made the necessary adjustments to meet these requirements. This includes adjusting the manuscript format, providing additional details regarding paper division and author contributions in the manuscript, supplying the ORCID iD for the corresponding author, supplementing a separate caption for each figure in the manuscript, as well as captions for Supporting Information files at the end of the manuscript, and other necessary adjustments as requested.

About the Role of Funder statement:The funders had no role in study design, data collection and analysis, decision to publish, or preparation of the manuscript.

**References:**

1.	Li S, Li M, Zhou N. Pricing and coordination in a dual-channel supply chain with a socially responsible manufacturer. PloS one. 2020;15(7):e0236099. doi: 10.1371/journal.pone.0236099.

2.	Yang D, Liu S, Zhang Z, Huang F, Wang J. Strategic interaction of original equipment manufacturers between outsourcing and purchasing in a quality-differentiated market. PloS one. 2022;17(1):e0262678. doi: 10.1371/journal.pone.0262678.

3.	Chen J, Liang L, Yao D-q. Factory encroachment and channel selection in an outsourced supply chain. International Journal of Production Economics. 2019;215:73-83. doi: 10.1016/j.ijpe.2018.05.003.

4.	Shi J. Contract manufacturer’s encroachment strategy and quality decision with different channel leadership structures. Computers & Industrial Engineering. 2019;137:106078. doi: 10.1016/j.cie.2019.106078.
